# Lysozyme Peptides as a Novel Nutra-Preservative to Control Some Food Poisoning and Food Spoilage Microorganisms

**DOI:** 10.1007/s12602-024-10226-2

**Published:** 2024-02-20

**Authors:** Adham M. Abdou, Dina A. B. Awad

**Affiliations:** Department of Food Hygiene and Control, Faculty of Veterinary Medicine, Benha University, Moshtohor, 13736 Kaliobeya Egypt

**Keywords:** Egg-white lysozyme, Antibacterial  peptides, Food poisoning, Food spoilage, Chemical preservatives, Natural food preservation

## Abstract

Foodborne illnesses and microbial food contamination are crucial concerns and still issues of great worldwide concern. Additionally, the serious health hazards associated with the use of chemical preservatives in food technology. Lysozyme (Lz) is an active protein against Gram-positive bacterial cell wall through its muramidase lytic activity; however, several authors could identify some antimicrobial peptides derived from Lz that have an exaggerated and broad-spectrum antibacterial activity. Therefore, a lysozyme peptides preparation (LzP) is developed to broaden the Lz spectrum. In this work, we investigated the potential efficacy of LzP as a novel Nutra-preservative (food origin) agent against some pathogenic and spoilage bacteria. Our results showed that LzP demonstrated only 11% of the lysozyme lytic activity. However, LzP exhibited strong antibacterial activity against *Escherichia coli*, *Salmonella enteritidis*, and *Pseudomonas* species, while *Salmonella typhi* and *Aeromonas hydrophila* exhibited slight resistance. Despite the lowest LzP concentration (0.1%) employed, it performs stronger antibacterial activity than weak organic acids (0.3%). Interestingly, the synergistic multi-component formulation (LzP, glycine, and citric acid) could inhibit 6 log_10_ cfu/ml of *E. coli* survival growth. The effect of heat treatment on LzP showed a decrease in its antibacterial activity at 5 and 67% by boiling at 100 °C/30 min, and autoclaving at 121 °C/15 min; respectively. On the other hand, LzP acquired stable antibacterial activity at different pH values (4–7). In conclusion, LzP would be an innovative, natural, and food origin preservative to control the growth of food poisoning and spoilage bacteria in food instead chemical one.

## Introduction

For both consumers and food technologists, the most pressing issues and challenges facing the food sector are food safety and quality. One of the most important concerns for food manufacturers is microbial contamination, which includes the presence of spoilage microorganisms in general and pathogens in particular. Therefore, one of the main priorities of food manufacturers and regulatory food agencies is to provide foodstuffs free of any microbial contaminants [1]. Interestingly, the frequency of foodborne illnesses in humans remains mostly unknown. Foodborne illnesses caused by pathogens present in various foods are considered a growing public health issue and encompass a wide spectrum of diseases [2]. Over the past ten years, the prevalence of foodborne microbial illnesses has increased significantly in most countries [3]. Food deterioration, food waste, and outbreaks of foodborne bacterial diseases continue despite the recent advancements in food preservation technologies [4].

The widespread use of chemical food preservatives is a result of their affordable cost and straightforward manufacturing procedure. Food preservation agents are vital and play a critical role in the battle against food deterioration. Nevertheless, the improperly used or prolonged consumption of chemical preservatives like sodium benzoate, sodium nitrate, sodium nitrite, and benzoic acid has been related to major health problems [5]. Additionally, regarding the negative impacts of commercial chemical preservatives on public health, individuals are also growing more interested in the replacement of chemical preservatives with natural alternatives drawn from natural systems [6, 7].

As consumer demand for natural ingredients and clean labels grows, it is anticipated that the industry will abandon chemical techniques of food preservation and switch to more natural alternatives, more particularly, nutra-preservatives (food origin preservatives) such as food-derived antimicrobial peptides and hydrolyzed food proteins [7, 8]. Many pathogens are incriminated in food contamination and harm the consumers, such as *Salmonella* spp., *Shigella* spp., *Micrococcus* spp., *Enterococcus faecalis*, *Escherichia coli*, *Bacillus licheniformis*, *Staphylococcus aureus*, *Campylobacter jejuni*, *Listeria monocytogenes*, *Yersinia enterocolitica*, *Escherichia coli 0157:H7*, *Vibrio parahemolyticus*, and *Clostridium botulinum*. Numerous studies have established the efficiency of antimicrobial peptides originating from food proteins and demonstrated effectiveness against a range of foodborne pathogens. Hence, these peptides can aid in food preservation in a natural and safe way [9, 10].

Lysozyme is a protein with a molecular mass of about 14 KDa that is found in many mucosal secretions (including saliva, tears, and mucus), tissues of plants, and animals. It is crucial for innate immunity, protecting against bacteria, viruses, and fungi. Due to its antibacterial qualities, it has long been the master important of numerous applications [11]. Historically, lysozyme is traditionally associated with chicken eggs. Egg white includes 11% proteins, 3.5% of which is lysozyme. As a result, this enzyme is one of the primary proteins in egg white where it serves as a defensive protein and feeds the developing embryo [12]. Lysozyme peptides (LzP) are a source of different biotechnological and health-promoting functions that can be used to create functional foods and nutraceuticals; besides, LzP showed a marvelous and broad spectrum antibacterial activity [7, 13].

The current study aims to investigate the efficiency of LzP on a variety of pathogenic and spoilage bacteria that could contaminate foodstuffs as well as study the variables impacting their antibacterial activity. Furthermore, we assess and compare the antibacterial properties of the multi-component food-grade antibacterial mixture compared to employing LzP alone. Additionally, food is typically subjected to a variety of processing methods so, we characterize the antibacterial efficacy of lysozyme peptides after its exposure to different food treatments such as spray drying, boiling, freezing, and different refrigerated storage conditions. Aiming to find an effective, safe, and more acceptable natural bio-preservative formulation was an alternative approach for controlling undesirable bacteria in food.

## Materials and Methods

### Microbial Strains and Culture Conditions

Commercial lysozyme powder derived from hen egg white (Lz) was received from Wako Chemicals (Osaka, Japan). Porcine pepsin A was supplied from Sigma-Aldrich (Meguro-ku, Tokyo, Japan). Test model indicator microorganisms for antibacterial assays, *Escherichia coli* K-12 (IFO 3301), *Salmonella enteritidis* (IFO 3313), and *Salmonella typhimurium* (ATCC 14028) strains were received from the Institute of Fermentation (Osaka, Japan). *Pseudomonas aeruginosa* (ATCC 27853), *Pseudomonas fluorescence* (ATCC 17386), and *Aeromonas hydrophila* (ATCC 7965) strains were obtained from the American Type Culture Collection (Rockville, MD, USA). Cultures were maintained in trypticase soy broth (TSB) with 15% glycerol at 20 °C. Brain–heart infusion (BHI) broth, trypticase soy broth (TSB), and nutrient agar were provided from Difco Laboratories (Detroit, MI, USA). The microbial substrate of Lz, *Micrococcus lysodeikticus*, and other chemical organic food additives including propionic acid, glycine, citric acid, potassium sorbate, and sodium benzoate were of food grade purchased from Sigma–Aldrich (St. Louis, MO, USA).

### Preparation Lysozyme Peptides

Lysozyme peptides powder (LzP) was obtained from Pharma Foods International Co., Ltd. (Kyoto, Japan). It was produced by partial enzymatic hydrolysis from egg white lysozyme using pepsin enzyme. The LzP powder is a mixture composed of 50% active peptides and 50% glycine. It was dissolved in 1.0% wt./vol in sterilized distilled water. The solution was gently stirred to avoid foaming, filtered, and kept refrigerated as a stock solution at 4 °C [7].

### Hydrolytic Activity

To examine the residual lytic activity of treated LzP in comparison to untreated intact Lz, the turbidimetric approach depending on the bacterial substrate *Micrococcus lysodeikticus* was used according to the previously published method [14]. The turbidity of three mixtures, each containing either 1.9 ml of *Micrococcus lysodeikticus* was mixed in 50 mM potassium phosphate buffer (pH 6.2) with 100 µl of solutions of LzP, Lz, and the third was potassium phosphate buffer only (50 mM, negative control). All were tested for turbidity using a spectrophotometer apparatus (SmartSpec-3000, Bio-Rad, USA in origin). The drop in suspension absorbance measurement at 450 nm at 25 °C was used to quantify the lysis of *Micrococcus lysodeikticus* cells. As a percentage of the activity of the untreated Lz, the LZP enzymatic activity was expressed.

### Antimicrobial Activity

The antibacterial liquid broth technique was used to assess the antimicrobial activity, according to a previous study [15]. Aliquots of mid-logarithmic phase bacterial suspension that had been grown in brain heart infusion (BHI) broth were harvested by centrifugation, washed by sterile saline, and resuspended trypticase soy broth (TSB), adjusted to a final bacterial concentration of 5 log_10_ cfu/ml of cells, and then were mixed with an equal volume of the medium containing test intact protein (Lz) or its peptides. The LzP-free controls were incubated (act as control). The suspensions were incubated at the given temperature for 4 h, serially diluted in physiological saline, and disseminated on nutrient agar. Colony-forming units were obtained after the plates were incubated at a certain temperature for a pre-determined time. The formula used to calculate the killing power was as follows:

Killing power% = (log_10_ Ctrl − log_10_ T)/ log_10_ Ctrl X100.

where Ctrl and *T* are cfu/ml of the control and treated groups; respectively. Except as noted otherwise, all assays were performed in triplicate, and the results represent the average of three independent trials.

### Sodium Dodecyl Sulfate–Polyacrylamide Gel Electrophoresis (SDS-PAGE)

The protein profiles of the lysozyme peptic digests were performed using SDS–PAGE Tris-Tricine ready gels (Bio-Rad Laboratories, Hercules, CA, USA). Samples were diluted in Tris-Tricine sample buffer (BioRad, USA). Electrophoresis was carried out at 100 V, for 3 h, at room temperature, in Tris-Tricine SDS running buffer. Rather than using the standard marker that follows, silver nitrate was utilized to visualize and stain the protein bands instantly.

### Characterization of LzP

#### Effect of Temperature

In a test tube, 4.5 ml of saline was combined with 0.5 ml of LzP. Then, each test tube was covered with paraffin oil to prevent evaporation and then heated at different temperatures for various storage times (100 °C/30 min; 121 °C/15 lbs/15 min; 4 °C /30 d; −20 °C/ 7 d; and freeze dry). The preparations utilized in the previously described liquid broth assay to evaluate its antibacterial effectiveness against *Escherichia coli* [16].

#### Effect of pH

The effect of pH was investigated by adding 0.5 ml of LzP into 4.5 ml of nutritional broth at various pH levels (4 to 8); then, the mixtures were incubated for 30 min at 37 °C. *Escherichia coli* resistance testing was performed on each LzP sample that had been subjected to a variety of pH levels [16].

### Comparing LzP Efficacy with Organic Weak Acids Against *Escherichia coli*

Different concentrations of frequently employed chemical organic food preservatives, such as glycine, citric acid, propionic acid, potassium sorbate, and sodium benzoate at a concentration of 0.3% were incubated with *E. coli* at 37 °C/4 h in comparison to LzP at concentration 0.1% in presence of negative control, which contained *E. coli* bacterial suspension only. After the plates had been incubated at 37 °C for 18 h, colony-forming units were collected.

### Formulation of Multi-component Antibacterial Mixture Against *Escherichia coli*

Different concentrations of LzP were used as one component of the antibacterial mixture. The multi-component of the antibacterial formulations was composed of LzP, glycine, and/or citric acid at different concentrations as shown in Fig. 4. Liquid broth assay, as mentioned previously, was used to test LzP and other formulations against *E. coli.*

### Statistical Analysis

The means and standard deviations of each set of data were displayed in triplicate. Using a one-way ANOVA, the significance of the differences was established. A difference was deemed statistically significant if the *p* value was less than 0.05.

## Results

### Antibacterial Activity of LzP Against Pathogenic and Spoilage Bacteria

**Fig. 1:**
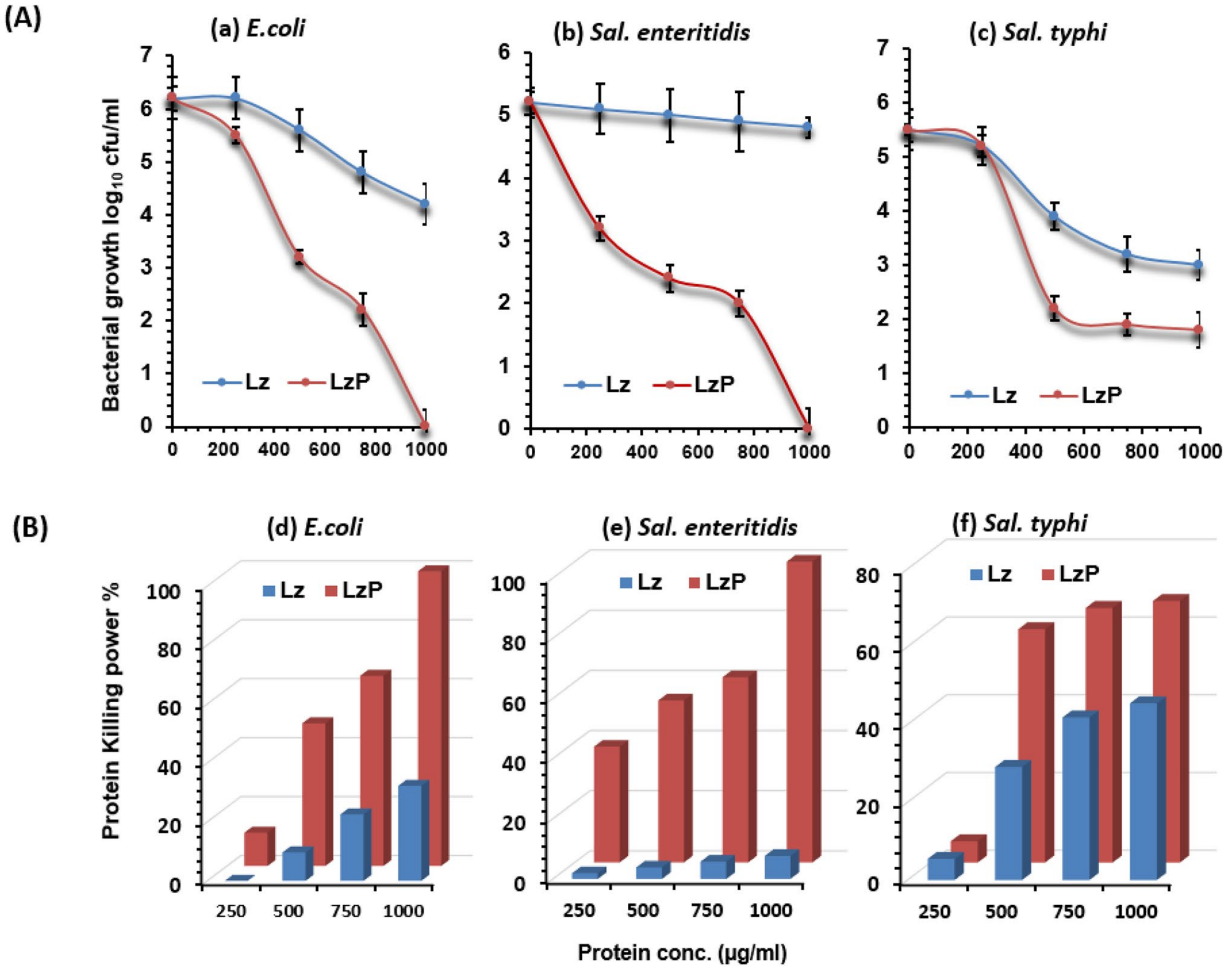
Antibacterial activity against food poisoning bacteria. **A** The antibacterial assay was performed against *E. coli*, *Salmonella enteritidis*, and *Salmonella typhimurium*, at different doses of lysozyme peptides (LzP) or intact lysozyme (Lz). **B** The killing power % at different concentrations. The assays were performed in mean for three replicates with standard error

Figure 1A showed the bacterial survival growth rate expressed as log_10_ cfu/ml against the most pathogenic foodborne tested bacteria in the presence of different concentrations (250, 500, 750, and 1000 µg/ml (*w/v*), of Lz and its peptic digested form (LzP) after specific incubation period for 4 h at 37 °C. It was evident that LzP exhibited higher antibacterial activity than Lz in a dose-dependent behavior against *E. coli*, *Sal. enteritidis*, and *Sal. typhi*. The higher LzP concentration (1000 µg/ml) could result in complete bacterial inhibition with 100% killing power against the most pathogenic bacteria *E. coli* and *Sal. enteritidis* as illustrated in Fig. 1B. Although *Sal. typhi* showed slight resistance, but a 3.7 ± 0.32 log_10_ cfu/ml growth reduction could be achieved by LzP at a concentration of 1000 µg/ml with killing power at 67.27%.

**Fig. 2:**
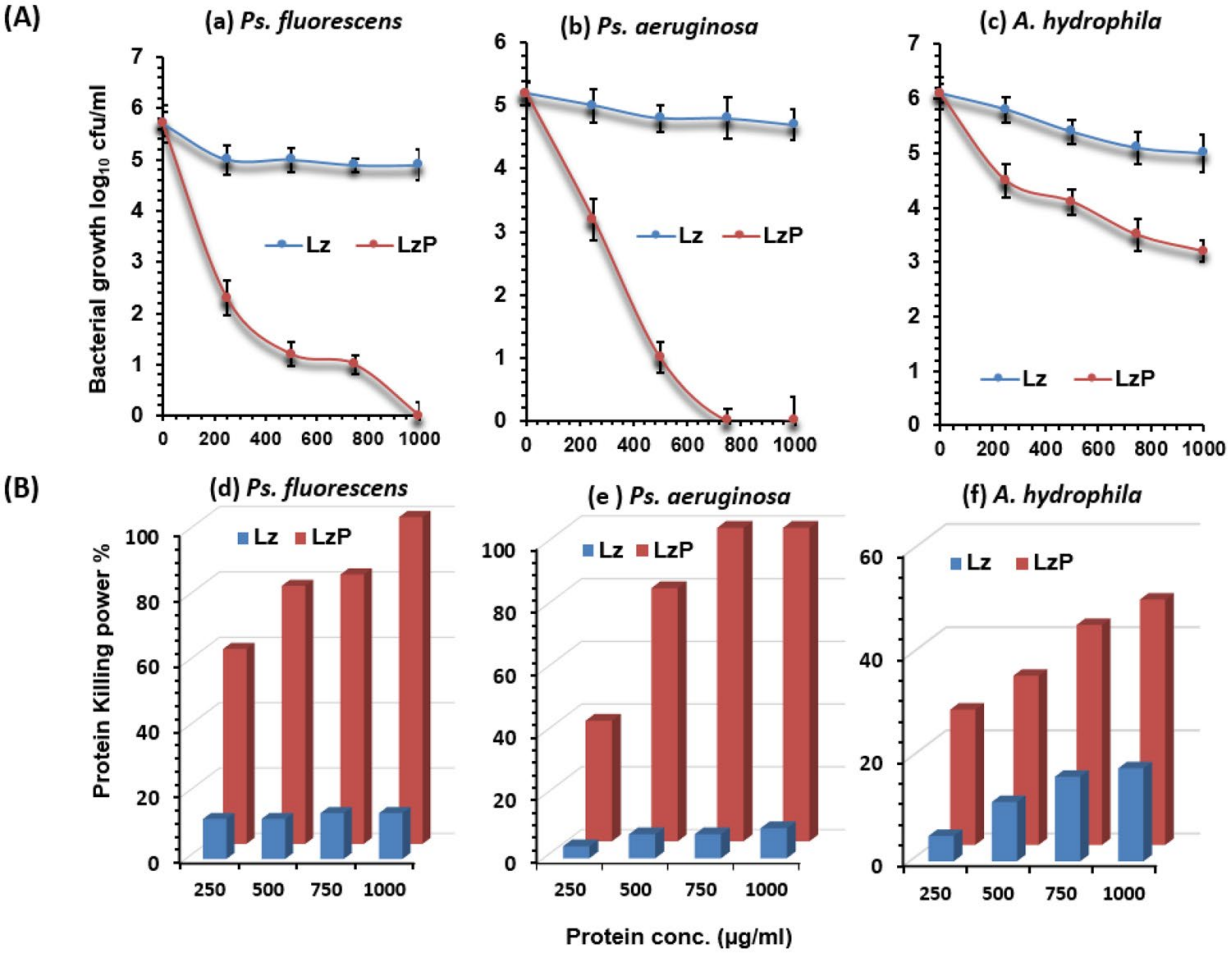
Antibacterial activity against food spoilage bacteria. **A** The antibacterial assay was performed against *Pseudomonas fluorescence*, *Pseudomonas aeruginosa*, and *Aeromonas hydrophila* at different doses of lysozyme peptides (LzP) or intact lysozyme (Lz). **B** The killing power % at different concentrations. The assays were performed in mean for three replicates with standard error

Figure 2A demonstrated the bacterial survival rate expressed as log_10_ cfu/ml against some indicator spoilage bacteria in the presence of different concentrations (250, 500, 750, and 1000 µg/ml (*w/v*), of intact Lz and its digested form LzP for 4 h incubation time at 37 °C. There was a significant increase (*p* < 0.05) in the tested indicator bacterial growth, compared with Lz and LzP under similar conditions. It was worth noting that LzP performed a higher antibacterial activity than Lz in a dose-dependent response against *Ps. Fluorescens, Ps. Aeruginosa,* and *A. hydrophila.* Interestingly, LzP at concentrations 750 and 1000 µg/ml was effective against *Ps. aeruginosa*, and *Ps. fluorescence* resulted in 100% killing power as illustrated in Fig. 2B. It was reported that *Ps. aeruginosa* was highly sensitive followed by *Ps. fluorescence* when treated with LzP, meanwhile *A. hydrophila* showed slight bacterial resistance. At the time, Lz could reduce *A. hydrophila* growth rate 1.1 ± 0.34 log_10_ cfu/ml with a killing power of 18.03%, LzP could reduce 2.9 ± 0.19 log_10_ cfu/ml with a killing power of 47.54% at a concentration 1000 µg/ml.

### Antibacterial Efficacy of LzP Compared with Organic Acids Against *Escherichia coli*

**Fig. 3:**
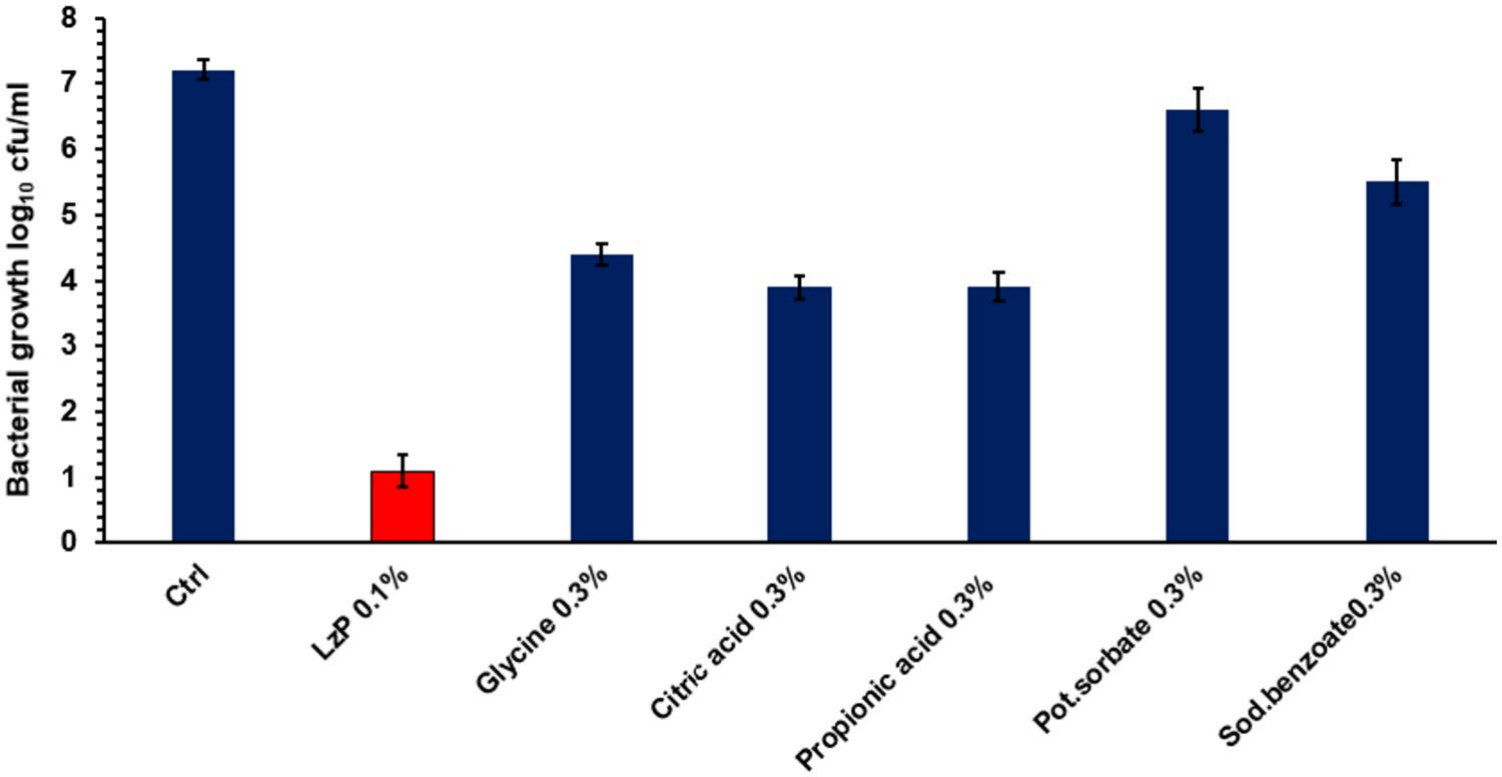
Comparative study between LzP and other chemical organic food preservatives commonly used in food preservation against *E. coli*. The result expressed as a mean value of three replicates ± standard error

In the food industry, synthetic chemical additives are used to improve the characteristics and properties of processed foods and include antimicrobial preservatives (glycine, citric acid, propionic acid, potassium sorbate, and sodium benzoate). Figure 3 showed the antibacterial activity of LzP and many widely utilized chemical weak organic acids used as food preservatives against *E. coli*. Despite utilizing a higher concentration of weak organic acids (0.3%) than LzP (0.1%) in the antibacterial assay, they performed less antibacterial impact on *E. coli* survival growth rate.

During the 4-h antibacterial assay incubation time, the most powerful weak organic acids (citric acid and propionic acid both at a concentration of 0.3%) were able to reduce the *E. coli* survival growth rate to roughly 3.9 ± 0.18 log_10_ cfu/ml, which is nearly half of the control value 7.2 ± 0.22 log_10_ cfu/ml. On the other hand, LzP 0.1% showed the ability to reduce *E. coli* survival growth to a level of 1.1 ± 0.24 log_10_ cfu/ml.

### Antibacterial Activity of LzP and Its Formulations

**Fig. 4:**
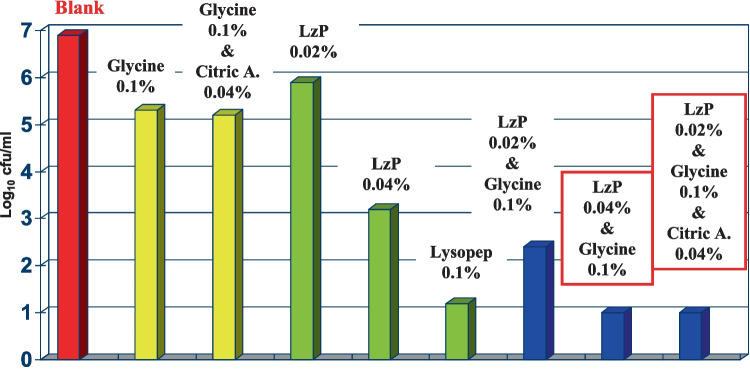
Antibacterial study of various multicomponent food preservatives against *E. coli*. The result expressed as a mean value of three replicates ± standard error. The letters (a, b, c, d, e) showing significance (≥ 0.05). **A** Weak organic acids, **B** LzP at different concentrations, and **C** mixture formulation between LzP and weak organic acids

The antibacterial activity of commonly used organic acids or even its combination formulation exhibited slight bactericidal activity upon incubation with *E. coli* as illustrated in Fig. 4A. There was no significant difference between the antibacterial effect of glycine (0.1%) or its combination with citric acid in a ratio (0.01:0.04%) when compared with control *E. coli* group. The synergistic antibacterial effect of the formulations made of LzP and organic acids, namely, glycine and citric acid were studied using liquid broth antibacterial assay. We added combinations of LzP and various organic acids to the *E. coli* bacterial suspension and then incubated it for a specific time at 37 °C/4 h after that the viable bacterial cell counts (cfu/ml) were enumerated as displayed in Fig. 4C. The LzP could inhibit *E. coli* survival rate in a concentration-dependent rate as the following, the highest LzP concentration 0.1% could decrease *E. coli* growth to 0.91 ± 0.31 log_10_ cfu/ml followed by LzP concentrations 0.04%, and then 0.02% that could reduce the bacterial count to 3.2 ± 0.29 and 5.7 ± 0.32 log_10_ cfu/ml; respectively, when compared to *E. coli* growth control which could reach 6.9 ± 0.36 log_10_ cfu/ml during the antibacterial assay as in Fig. 4B.

Upon LzP 0.04% and glycine 0.1% being formulated, a potent powerful combination is created that could reduce the *E. coli* growth to a lower detectable level of 0.9 ± 0.12 log_10_ cfu/ml. It was observed that the addition of citric acid 0.04% to the formulation (LzP 0.02%, and glycine 0.1%) did not improve the formula’s synergistic antibacterial power. On the other side, using glycine 0.1% or even formulated with citric acid 0.04% is less antibacterial effective as they could reduce the bacterial survival rate to 1.6 ± 0.15 log_10_ cfu/ml in comparison with *E. coli* growth control at 6.9 ± 0.36 log_10_ cfu/ml.

### Stability of LzP Antibacterial Activity

#### Heat Stability

The LzP’s antibacterial activity was evaluated for its stability at various degrees of temperatures and storage time on a model strain, *E. coli.* Survival rate after incubation for 4 h then the results calculated as inhibitory percent are presented in Table 1. Strikingly, we found that temperature and storage time had a greater impact on LzP antibacterial activity against *E. coli*. Treated LzP at 100 °C for 30 min resulted in maximum stability and the percentage of bacterial growth inhibition reached approximately 95%. Meanwhile, LzP subjected to autoclaving at 121 °C/15 lbs/15 min lost 67% of its antibacterial activity. On the contrary, the antibacterial activity of LzP was relatively resistant to different degrees of thermal storage conditions at 4 °C/30 d, −20 °C/7 d, and freeze drying as the antibacterial activity maintained 100% compared with the non-heat-treated LzP (Table 1).

**Table 1:** Effect of temperature and storage incubation time on the antibacterial activity of Lysozyme peptides (LzP) at different conditions against *E. coli*

Treatment	Antibacterial activity (%)
No treatment 100 °C/30 min 121 °C/15 lb in^−2^/ 15 min 4 °C/ 30 days −20 °C/ 7 days Freeze-dried	100 95 33 100 100 100

#### pH Stability

**Fig. 5:**
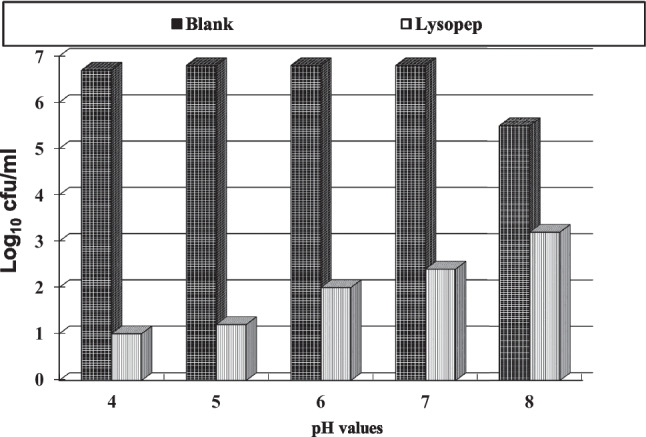
pH stability of LzP antibacterial activity against *E.coli* at various pH values. The result expressed as a mean value of three replicates ± standard error

Data in Fig. 5 showed the antimicrobial stability of LzP’s efficacy at different pH values, ranging from 4 to 8. It was observed that the antibacterial activity gradually decreased with increasing the pH values. Optimal antibacterial stability was observed at pH 4.0 to 7.0. LzP at a concentration of 1000 µg/ml showed about sixfold *E. coli* bacterial growth reduction. Growth patterns of *E. coli* in the absence of LzP was varied according to the pH value; its optimum growth rates were at pH 4–7 reaching 6.9 ± 0.36 log_10_ cfu/ml, whereas, by increasing the pH value to the alkaline side, *E. coli* showed a slight inhibitory decrease in the growth rate reached to 5.5 ± 0.23 log_10_ cfu/ml.

#### Lytic Activity and SDS-PAGE

**Fig. 6:**
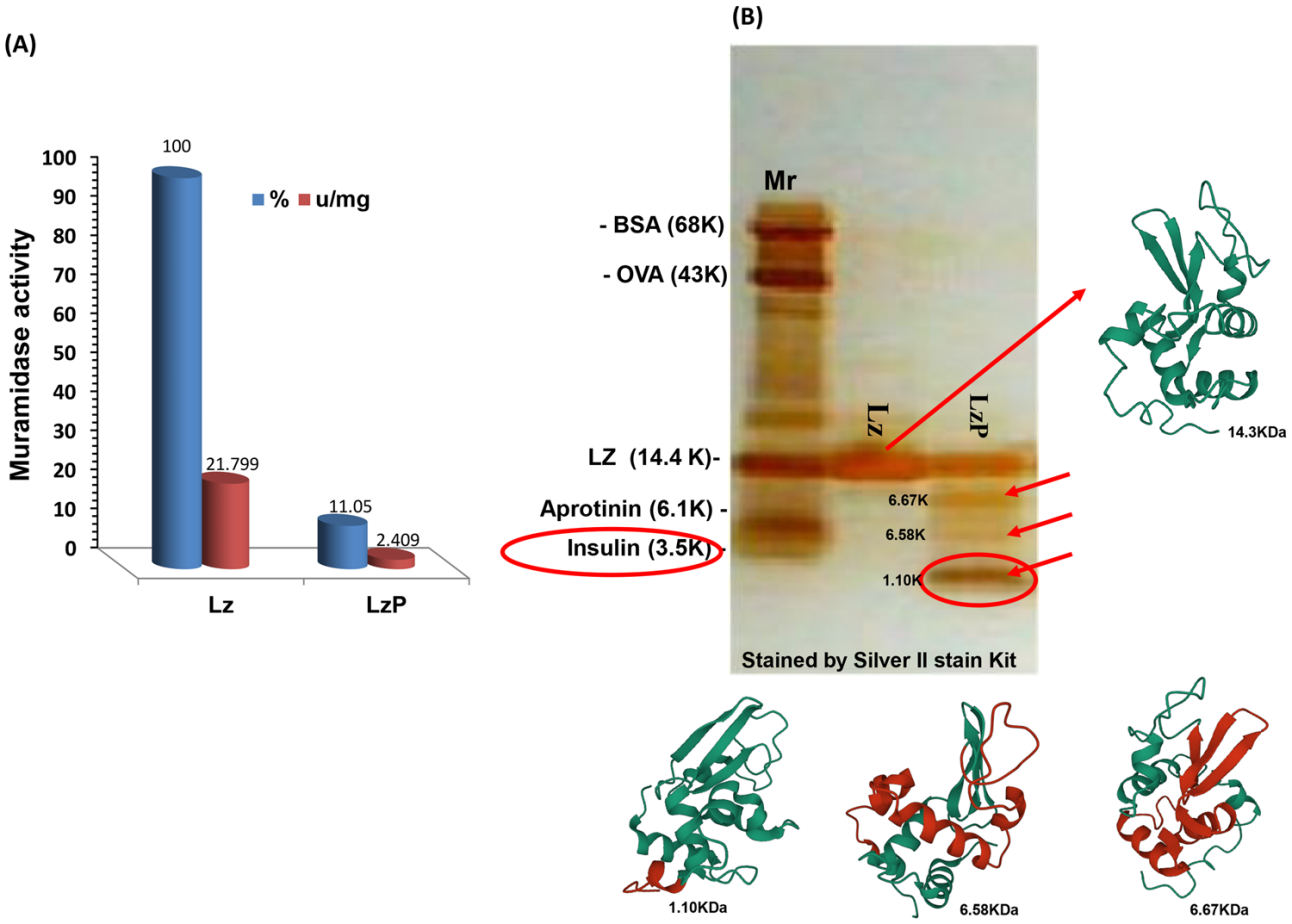
**A**, **B** Lytic activity of intact Lz and LzP. **C** SDS-PAGE stained by silver stain showing LzP. According to Protein Data Bank, we can predict the pepsin cleavage site on lysozyme molecule with red color

Figure 6A and Table 2 revealed that LzP only had 11.05% (2.409 u/mg) of the lytic activity of the untreated Lz (which is 100%: 21.799 u/mg). Although LzP lost about 89% of lytic activity, it demonstrated potent antibacterial action against *E. coli* compared to intact Lz.

**Table 2:** Muramidase activity of lysozyme and LzP

	Lz	LzP
Muramidase activity (%)	100.00	11.05
Muramidase activity (µ/mg)	21.799	2.409

SDS–PAGE of the peptic Lz digests was screened using Tris-Tricine ready gels with 16.5% acrylamide stained by silver stain illustrated in Fig. 6C. Clear band with molecular weight parallel to Lz at 14.4 KDa. Other small molecular weight peptide fragments appear corresponding to 7.36, 5.43, and 1.04 KDa also produced.

## Discussion

Foodborne illnesses caused by various microbes present in foods are considered a necessary emerging public health issue and encompass a wide range of food poisoning diseases [2].

Food additives such as benzoate salts (sodium or potassium), carrageenan, and tartrazine are chemicals that are added to improve food organoleptic qualities and prevent spoilage. Most of synthetic artificial chemicals utilized as food additives are associated with negative health hazards, causing malignancies and mutagenic respiratory disorders [17].

Food proteins can be hydrolyzed to small peptides by three approaches, enzymatically (using proteolytic enzymes extracted from either plant origin or microbial origin), hydrolyzed with digestive enzymes (simulating gastrointestinal digestion in humans), or fermented using bacterial starter cultures to yield functional bioactive peptides. In the first approach using the enzymatic hydrolysis technique, the target intact parent protein is subjected to an enzymatic process at a certain pH buffer and temperature within a specific time. The benefits of this approach include ease of scaling up and a generally controlled, clean, faster reaction time than microbial fermentation which may produce other compounds [18]. Within the protein sequence, various bioactive peptides are encoded that serve as functional active ingredients [19]. The peptides generated can be more precisely controlled when intact parent proteins are hydrolyzed by enzymes outside of the gastrointestinal tract. These can then be searched individually or in combination for biological functions and potential applications that diverge from those of the parent proteins [13]. Muramidase, a lysozyme one of the proteins derived from hen egg albumen that belongs to the family of glycosidic hydrolases catalyzes the lysis of the β (1–4) link between N-acetylglucosamine and N-acetylmuramic acid in bacterial cell walls. Its primary structure is a single polypeptide chain with 129 amino acids, as illustrated in the Protein Data Bank (PDB code 1HEW) displayed in Fig. 6C. Usually, it disintegrates into a compact, spherical secondary structure with a surface slit [20, 21]. Pepsin at pH 4.0 primarily breaks down peptide bonds that contain aromatic hydrophobic amino acids, with Phe, Trp, and Leu residues providing the best cleavage sites [22]. It is necessary to point out that a previous study [23] could identify and locate the peptide fragments using MALDI-TOF–MS analysis that supports our validated SDS-PAGE findings in Fig. 6C. It was noted that incomplete peptic hydrolysis of Lz produced more active, smaller peptides with molecular weights of 7.3, 5.4, and 1.04 KDa and left 60% of the Lz protein intact that referring to LzP. Although Lz’s catalytic activity is more crucial for bacterial growth control, LzP has less lytic activity (11.05%) (Fig. 6A and Table 2). Even though LzP could perform more potent antibacterial activity which is attributed to the stronger generated peptides.

Our study was with particular emphasis on exploring the antibacterial activity of LzP on different pathogenic and spoilage bacteria as well as the variables influencing its antibacterial activity assay efficacy. *Escherichia coli* species and *Salmonella* species are the most prevalent pathogens, whereas *Pseudomonas aeruginosa* is one of the opportunistic bacteria among the most prevalent pathogens encountered in different food deterioration in a wide range of various vegetables, meat, and milk products [24].

According to our findings, the LzP effectively combats the survival of *E. coli* and *Sal. enteritidis* but *Sal. typhimurium* exhibits a small amount of resistance which is frequently associated with food poisoning with virulence characteristics and drug resistance, which may be to blame for this resistance. The primary mechanisms of resistance are altered lactamase and penicillin-binding proteins, decreased permeability of the outer membrane, and activation and synthesis of efflux pumps [25, 26].

A previous study revealed the isolation of *Pseudomonas* spp. from different milk and milk products including non-thermal treated milk, Karish cheese, yogurt, and ice cream [27]. Both *Ps. aeruginosa* and *Ps. fluorescence* were more vulnerable to LzP. While our lysozyme peptides exhibit potent bactericidal activity against different *Pseudomonas* spp., selenium, and chitosan nanoparticles at various concentrations in a dose-dependent mechanism could partially inhibit bacterial growth after five days cooling storage as previously reported [28]. On the other hand, *A. hydrophila* showed slight resistance; where LzP was only 47% lethal at a concentration of 1000 µg/ml. *A. hydrophila* is a Gram-negative, facultatively anaerobic, oxidase-positive, opportunistic marine pathogen causing gastroenteritis. It produces a variety of virulent factors as enterotoxins and lytic enzymes [29]. It has been isolated from various food items such as meat, fish, milk, and vegetables. However, numerous research revealed that this opportunistic pathogen is resistant to commercial antimicrobials. Recognition of *A. hydrophila* as an anaerobe is required to make the antibacterial activity of LzP decrease due to the need for particular growing conditions during the antibacterial assay [30]. Generally, to prevent microbial growth in the food industrial sector, chemical preservatives like benzoate, propionate, sorbate, nitrate, and sulfites are frequently utilized [31]. Recently, it has been observed that synthetic preservatives have raised many health concerns and issues. As consumers are becoming more conscious of the relationship between health issues and their diet, consumer awareness is increasing about the synthetic-based antimicrobials in food formulations. Due to worries over these compounds' long-term use, which results in liver damage, asthma, numerous allergic reactions, tumors, and even cancer, therefore, most people are turning to natural antimicrobials [32]. Consequently, the use of synthetic preservatives has negative effects on human health, and food researchers and consumers discourage their usage. However, numerous studies have demonstrated a link between the overuse of synthetic food additives is related to gastrointestinal, respiratory, dermatological, and neurological adverse reactions [32]. Due to these public health risks caused by weak organic acids, it is imperative to find natural antimicrobials that can effectively combat these organic acid-based public health risks.

Using a liquid broth antibacterial experiment, the pre-screening effects of food-grade weak organic acids on *E. coli* survival in comparison to LzP were studied. *E. coli* is less sensitive to the effect of organic acids although 0.3% of organic acids were used as opposed to 0.1% of LzP, we observe *E. coli* had less sensitivity to the effect of organic acids as Gram-negative bacteria are typically less susceptible to weak acids action because the bacterial protective outer membrane, which serves as a protective barrier to organic acids action [33]. The LzP was tested for antibacterial activity against *E. coli*. Then formulation of LzP, glycine, and citric acids was tested in the current study. According to earlier studies, organic acids are frequently utilized as food preservatives due to their antibacterial qualities. Particularly, the undissociated form of the acid that can freely diffuse past the membrane of microbes and into their cell cytoplasm is what weak organic acids’ antimicrobial activity depends on. The acid will dissociate, and anions will collect once inside the cell, where the pH is almost neutral, inhibiting cell enzymes (decarboxylases and catalases) and nutritional transport mechanisms [34]. Contrarily, antimicrobial LzP functions as membrane-disrupting antibacterial agents that engage with the bacterial membrane to create pores, which ultimately cause bacterial death [35]. Different interactions may arise when antibacterial agents are combined, leading to a variety of effects that could be additive, antagonistic, or synergistic [36]. Combining antibacterial agents produces stronger effects that boost antibacterial activity and enable the use of lower dosages of chemical organic antibacterial agents that are safe for use in food. According to the findings of our experiments, LzP activity has stronger antibacterial activity than weak organic acids. However, in the time-kill assay, neither synergistic effects nor additional value between LzP and citric acid was observed. There was no discernible difference between glycine 0.1% (5.45 ± 0.45 log_10_ cfu/ml) or when coupled with citric acid 0.04% (5.11 ± 0.36 log_10_ cfu/ml). There were no appreciable differences observed between LzP 0.1% alone (0.9 ± 0.31 log_10_ cfu/ml) and when combined with glycine and/or citric acid. Otherwise, it would be beneficiary as we use LzP at low concentrations with glycine and/or citric acid, this may be of importance to reduce the preservation costs. The slight difference in bacterial inhibition may be attributed to the LzP mechanism creating pores or tunnels in the cell membrane making it easier for organic acids to pass inside the bacterial cells. Our results were in line with the previous study [37] which demonstrated that Lz and citric acid together had no added benefit.

The current food processing methods and storage can affect the efficacy of natural antimicrobials. So, comparative studies of the relative impact of LzP, such as pH, temperature, and storage time on the growth survival of *E. coli*. To address this issue, we compared the antimicrobial action of treated LzP on *E. coli* under several circumstances. It is crucial to identify how thermal storage conditions and pH levels affect the antibacterial stability of LzP because many food-related factors can completely or partially affect the function of these compounds.

High-pressure treatment and autoclaving pressure affect the different forms of protein structure the secondary, tertiary, and quaternary resulting in reversible alterations with induction permanent denaturation [38]. Proteins undergo irreversible denaturation supposedly to be due to the breakdown of the hydrogen bonds that stabilize and support the secondary structure [39]. This could account for why autoclaving LzP results in a significant decrease in its antibacterial effect. The highly inhibitory effect of LzP during boiling for 30 min or cooling storage gives it a great opportunity and a vital role in foods undergoing thermal processing.

In general, most pathogenic bacteria can typically grow in a pH range of 4.0 to 9.0, with the optimum pH range from 6.50 to 7.50 [40]. The influence of pH values on LzP antibacterial stability was considerable, with a weak acidic zone (pH 4.0–6.0), while less stable at higher pH over 6 (alkaline side).

Finally, the findings presented in this study add fresh knowledge about the ideal circumstances in which antibacterial peptides (LzP) execute their most effective antibacterial activity and offer an intriguing possibility for the prospective use of antibacterial peptides (LzP) as an effective, novel, food origin preservative (nutra-preservative), safe, and natural food preservative delegate is offered by the study’s findings. We recommend future studies to isolate and identify the peptide sequence for industrial food applications.

## Conclusion

Due to the adverse effects of industrial synthetic chemical preservatives and their carcinogenicity and toxicity for humans, the debates have increased on using natural preservatives in addition to the progress in foodborne illness outbreaks. It is therefore crucial to find alternatives to conventional food antimicrobials. In this work, we present a viable alternative by simply using lysozyme peptides using pepsin (LzP). It performs a potent antibacterial effect against most spoilage such as *Pseudomonas* spp. and foodborne pathogens such as *E. coli* and *Sal. enteritidis*. LzP at low concentrations acquired more potent antibacterial activity than using weak organic acids (glycine and citric acid) which are commonly used in the food-processing industry. In addition, it was further found that the formulated mixture of food-grade weak acids to LzP did not add more synergistic antibacterial value. It was found that LzP was effective at a wide range of pH (4–8) and maintains good stability against thermal processing at 100 °C/ 30 min in addition withstand the storage cooling conditions. The electrophoretic patterns revealed 60% intact lysozyme and small molecular weight peptides (7.3, 5.4, and 1.04 KDa) which contributed to the antibacterial activity. We, therefore, suggest the prospective use of LZP as an eco-friendly approach to food preservation.
